# Pyoderma Gangrenosum Associated With Seronegative Arthritis: A Rural Tertiary Care Experience

**DOI:** 10.7759/cureus.14770

**Published:** 2021-04-30

**Authors:** Tanishq S Sharma, Dr Roy Mali, Hardil P Majmudar, Saptak P Mankad

**Affiliations:** 1 Medicine, Shree Krishna Hospital, Anand, IND; 2 Internal Medicine, Pramukhswami Medical College, Anand, IND; 3 Medicine, Pramukhswami Medical College, Anand, IND; 4 Internal Medicine, Shree Krishna Hospital, Anand, IND

**Keywords:** seronegative arthritis, pyoderma gangenosum, symmetric bilateral lesions, non-healing ulcers, polyarthritis

## Abstract

Pyoderma gangrenosum (PG) is a rare ulcerating inflammatory skin condition, the cause of which remains idiopathic. A 35-year-old female presented to our outpatient department (OPD) with initial findings of multiple non-healing ulcers that developed suddenly with a symmetric bilateral pattern on the dorsal aspect of hands, elbows, and inner knees. Lesions initially developed as a pustule that progressed to burst and leave behind a raw area that turned into an ulcer. Polyarthralgia preceded these symptoms. Extensive investigations were done for the varied differentials that were postulated; this led to finally declaring PG associated with seronegative arthritis as a diagnosis of exclusion. Initial treatment with antibiotics showed little results, and thus, the patient was started on a systemic corticosteroid which proved to be successful.

## Introduction

Pyoderma gangrenosum (PG) is a rare, painful, ulcerative, chronic cutaneous pathology that is often found associated with other systemic conditions. It was first clinically described by Brunsting in 1930 as chronic cutaneous ulcers with bluish, serpiginous borders, and undermined edges with granulations present which were based on the stage of activity [1]. He also suggested a systemic association for the same [1]. Inflammatory bowel disease, arthritis, monogammopathies, hepatitis, myeloproliferative disorders are frequently associated with PG. The patient usually visits the clinic for complaints relating to these systemic disorders from where they are referred for this associated skin pathology. The exact cause of this rare condition is unknown. Patients develop chronic, solitary, or multiple ulcers on extremities which are often found to be present due to previous trivial trauma to the ulcer site known as pathergy [2]. PG is mainly a diagnosis of exclusion and can be diagnosed clinically but confirmed only after a skin biopsy. Multiple treatment modalities have been suggested but mainstay therapy includes systemic corticosteroids, immunosuppressants like cyclosporine, and azathioprine among others. Treating the underlying systemic condition is necessary. Here, we report a patient with PG associated with seronegative arthritis.

## Case presentation

**Figure 1:**
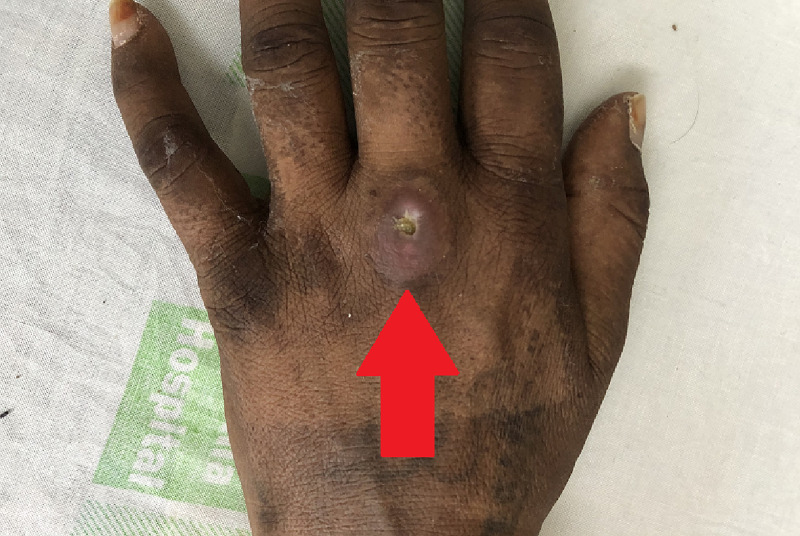
Ulcer on the Dorsal Aspect of Hand

**Figure 2:**
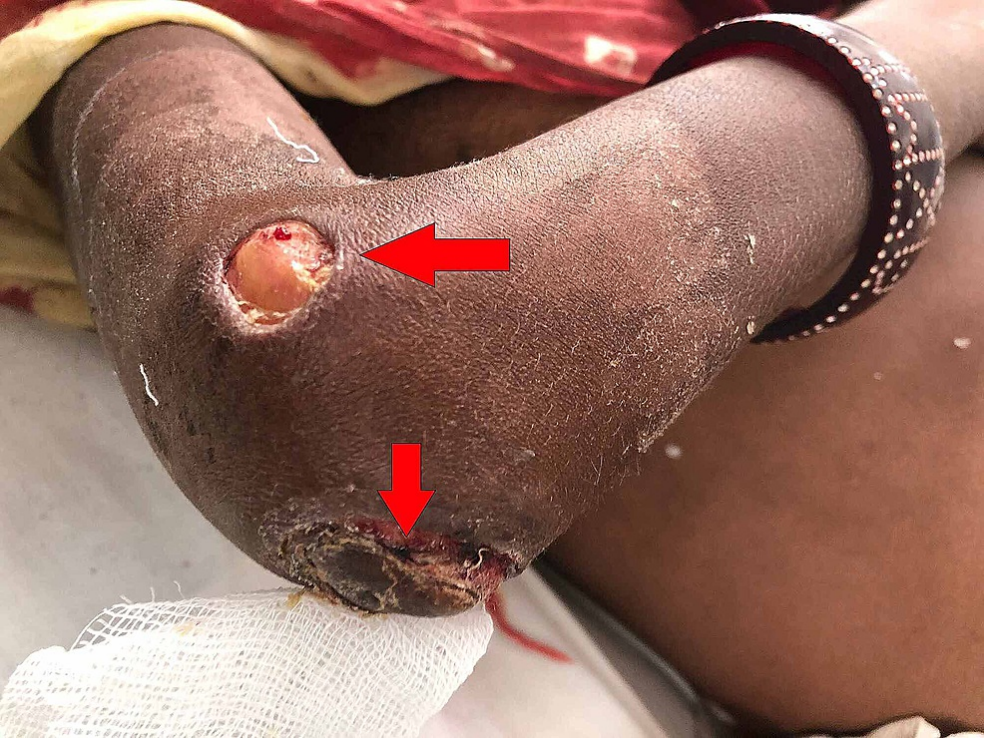
Ulcer on Postero-Lateral Aspects of Elbow

**Figure 3:**
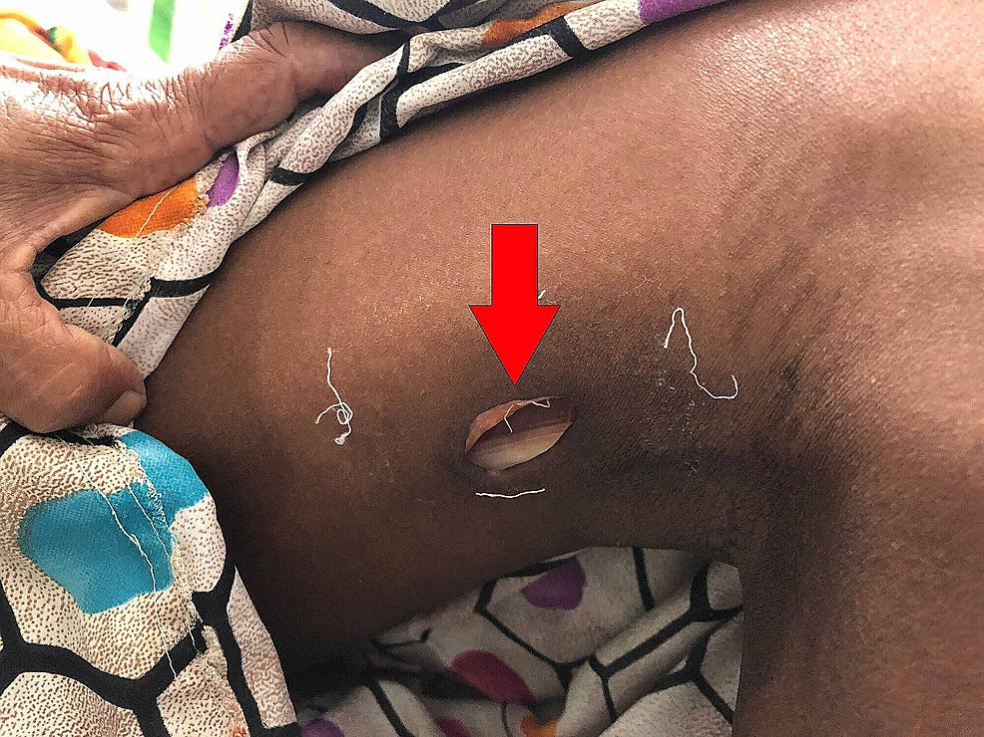
Ulcer on Inner Aspect of Knee

A 35-years-old female presented to our outpatient department (OPD) for the assessment of multiple non-healing ulcers present bilaterally on the dorsal aspect of hands, posterolateral aspect of elbows, and inner knees (Figures 1-3). The patient had severe pain at the sites of the ulcer which was present throughout the day. The patient first noticed a hard-elevated lesion on her left elbow two months ago. The lesion first began as a small pustule. It increased in size and became pus-filled to form a blister which later on burst to leave behind a raw area that turned into an ulcer. Several such lesions developed gradually on other parts of her body that progressed similarly. The lesion progressed to her right elbow, advanced further to both knees, and then lastly to the dorsal aspect of both hands. All the lesions turned into painful pus discharging ulcers over time. The patient initially consulted a private practitioner and was prescribed antibiotics along with an analgesic. The patient was unresponsive to this treatment following which she was referred to our tertiary care centre. On initial examination, the lesions were painful that discharged pus. Margins were raised, irregular, and showed bilateral symmetry. A few differentials were suggested like cutaneous tuberculosis, vasculitis, deep fungal infection, and PG. To rule out cutaneous tuberculosis, investigations were done which turned out negative. Further, a skin biopsy from the left elbow ulcer was sent to rule out other differentials.

The biopsy report showed epidermal and dermal ulceration with necrosis, vascular proliferation, granular tissue formation with polymorphs, lymphocytes, plasma cells, and macrophages. The adjacent epidermis showed spongiosis, while there was mild hyperplasia and peri adnexal mixed inflammatory infiltration in the dermis. These histological features were consistent with the clinical diagnosis of pyoderma gangrenosum. Antinuclear antibody (ANA) profile and rheumatoid factor (RF) were done to trace an autoimmune origin as she had complaints of polyarthralgia which turned out negative. So we diagnosed her as a case of seronegative arthritis on the basis of her symptoms. Based on the clinical progression of the lesions, their pathergic response, resistance to antimicrobial therapy, negative acid-fast bacteria (AFB) staining, and skin biopsy findings along with the pre-existing seronegative arthritis we diagnosed her as a case of pyoderma gangrenosum. The culture sensitivity report of the pus suggested pseudomonas which was sensitive to piperacillin and tazobactam. The patient was started on oral prednisolone 40 mg (OD), injectable clindamycin (TDS), and piperacillin-tazobactam (TDS). The patient was under observation for five days and seemed to respond well to the therapy. She was discharged after one week with the guidance of home care and follow-up advice.

## Discussion

Pyoderma gangrenosum is a rare ulcerating inflammatory skin condition, the cause of which remains unknown. It may present as solitary or multiple lesions with the most common location being lower extremities. This disease affects young to middle age groups with a higher female preponderance than males [3]. Brunsting et al. pioneered the term “PYODERMA GANGRENOSUM” in the 1930s. During their period of observation, they established a possible relationship between underlying debilitating disease and cutaneous manifestations [1].

About 50% of PG patients have an associated systemic disease, mainly Inflammatory bowel disease, neoplastic disease, and arthritis [3]. Powell et al. in their study review of 86 patients found that up to 37% of PG patients had associated arthritis (PGA) [4]. Seropositive and seronegative arthritis were the two forms of arthritis usually associated with PG [3]. Our patient presented with seronegative arthritis associated with PG and this was a diagnosis of exclusion. Likely, the inflammatory process associated with arthritis (seropositive or seronegative) is causal to the ulcers experienced by the patient [3]. Pathergy (Induction of lesions following a trivial injury to the skin) is a common clinical feature seen amongst PG patients and this finding was supported in our patient with the presence of ulcerations at sites of IV line on bilateral dorsal aspects of the hands.

Our patient initially presented with a pustule that elevated forming a pus-filled blister. It later burst to leave behind a raw area of ulceration. A rather unique finding was the development of symmetrical lesions on bilateral aspects of upper and lower extremities on the dorsal aspects of both hands, elbow regions, and inner aspect of the knee joint. These findings were supported by a study by Callen who described that the initial lesion is usually a pustule followed by symmetrical central ulceration containing necrotic tissue, blood, and exudate. Furthermore, he established that this typical course in patients with seronegative rheumatoid arthritis not associated with inflammatory bowel disease is similar to that of PG [2]. 

Varied differentials have been established for ulcerative cutaneous lesions which include: (1) Infectious diseases such as tuberculosis, deep fungal infections, bacterial infections, viral infections like acquired immunodeficiency syndrome (AIDS), (2) vasculitis (3) insect bites (4) venous or arterial insufficiency, and (5) factitial ulcerations [2]. No diagnostic test is available for PG, and therefore, it requires extensive workup and is a diagnosis of exclusion. Our patient was initially a suspect of cutaneous tuberculosis and so was investigated for the same. Because of negative AFB stain and inconclusive Mantoux test, the differential was excluded and subsequently, a histopathological biopsy from the left elbow ulcer was sent which concluded positive findings in favor of PG, and a definitive diagnosis was established. Trent and Kirsner in their study established modalities for the usual evaluation of PG that involved aspects like basic history and general examination, wound and tissue cultures, biopsy for histology, complete blood counts, hepatic functions, vascular studies, occult testing of stool, serum protein electrophoresis, rheumatoid factor, and chest radiograph [5]. 

Weenig et al. in their study identified 95 patients with ulcers that resembled pyoderma gangrenosum. These ulcers were the manifestations of vascular occlusive or venous diseases, cancers, drug-induced, exogenous injuries, or other inflammatory conditions. Weening et al. emphasized the possibility of misdiagnosis of skin ulcers as PG. We also reiterate that unnecessary diagnosis can lead to complications associated with treatment or it delays the diagnosis of an alternative disease process. Extensive workup and exclusion of differentials formed the mainstay for an accurate evaluation of PG in our patient [6].

The treatment approach to PG involves multiple entities, before the initiation of treatment. It is necessary to exclude other diagnoses in particular infectious causes before starting systemic corticosteroids. Underlying systemic causes must also be adhered to on a priority basis as successful treatment of associated disease helps in healing the ulcer faster. Systemic corticosteroids are the drug of choice for topical resistant PG and indicated in doses of 40-80 mg daily or higher (60-120 mg) to induce remission. Doses are tapered once remission has been initiated, no new lesions develop, and existing lesions stop aggravating. It is yet not clear whether corticosteroids act via immunosuppression, anti-inflammatory, or other mechanisms [7].

Immunosuppressive agents like cyclosporine, azathioprine, mercaptopurine, etc., have been widely used as an adjunctive or alternative therapy for patients of PG in whom systemic corticosteroids were unsuccessful or had side effects. Cyclosporine has shown promising effects in the treatment of PG [7]. Our patient showed improvements with antibiotics and corticosteroids and was considered under review for the addition of immunomodulators in case of further deterioration. But our patient remained stable and considerable ulcer healing was observed.

## Conclusions

Diagnosis of PG comes with multiple hurdles because of its many differentials. A rather unusual finding encountered in this case was the presence and development of lesions in a symmetric bilateral pattern. The patient simultaneously developed polyarthritis which on investigations turned out to be seronegative thus portraying an impression of arthritis-induced PG. Since this is a diagnosis of exclusion, treatment is to be started only after excluding all other differentials. Due care has to be undertaken to prevent excessive surgical debridement or skin grafting to avert a pathergic response. Our patient was started on antibiotics, systemic corticosteroids, and other supportive medications after the exclusion of an infective differential and later on showed considerable improvement with corticosteroids and antimicrobial combinations leading to the cessation of ulcer spread and newer development. Re-epithelization was slower but a considerable improvement was observed in the patient.

## References

[1] Brunsting LA (1930). Pyoderma (echthyma) gangrenosum: clinical and experimental observations in five cases occurring in adults. Arch Derm Syphilol.

[2] Callen JP (1989). Pyoderma Gangrenosum and Related Disorders. Med Clin North Am.

[3] Charles CA, Bialy TL, Falabella AF, Eaglstein WH, Kerdel FA, Kirsner RS (2004). Poor prognosis of arthritis-associated pyoderma gangrenosum. Arch Dermatol.

[4] Powell FC, Schroeter AL, Su WP, Perry HO (1985). Pyoderma gangrenosum: a review of 86 patients. Q J Med.

[5] Trent JT, Kirsner RS (2001). Diagnosing pyoderma gangrenosum. Adv Skin Wound Care.

[6] Weenig RH, Davis MD, Dahl PR, Su WP (2002). Skin ulcers misdiagnosed as pyoderma gangrenosum. N Engl J Med.

[7] Chow RKP, Ho VC (1996 Jun). Treatment of pyoderma gangrenosum. JAAD.

